# Effect of Triptolide on Temporal Expression of Cell Cycle Regulators During Cardiac Hypertrophy

**DOI:** 10.3389/fphar.2020.566938

**Published:** 2020-09-04

**Authors:** Jing-Mei Li, Xi-Chun Pan, Yuan-Yuan Ding, Yang-Fei Tong, Xiao-Hong Chen, Ya Liu, Hai-Gang Zhang

**Affiliations:** ^1^ Department of Pharmacology, College of Pharmacy, Army Medical University (Third Military Medical University), Chongqing, China; ^2^ Institute of Medical Biology, Chinese Academy of Medical Sciences & Peking Union Medical College, Kunming, China; ^3^ Department of Pharmacy, Chongqing Traditional Medicine Hospital, Chongqing, China

**Keywords:** cardiac hypertrophy, cardiomyocyte, cell cycle, cyclin, cyclin-dependent kinase, triptolide

## Abstract

Adult mammalian cardiomyocytes may reenter the cell cycle and cause cardiac hypertrophy. Triptolide (TP) can regulate the expressions of various cell cycle regulators in cancer cells. However, its effects on cell cycle regulators during myocardial hypertrophy and mechanism are unclear. This study was designed to explore the profile of cell cycle of cardiomyocytes and the temporal expression of their regulators during cardiac hypertrophy, as well as the effects of TP. The hypertrophy models employed were neonatal rat ventricular myocytes (NRVMs) stimulated with angiotensin II (Ang II) for scheduled times (from 5 min to 48 h) *in vitro* and mice treated with isoprenaline (Iso) for from 1 to 21 days, respectively. TP was used *in vitro* at 1 μg/L and *in vivo* at 10 μg/kg. NRVMs were analyzed using flow cytometry to detect the cell cycle, and the expression levels of mRNA and protein of various cell cycle regulators were determined using real-time PCR and Western blot. It was found NRVM numbers in phases S and G_2_ increased, while that in the G_1_ phase decreased significantly after Ang II stimulation. The mRNA expression levels of p21 and p27 increased soon after stimulation, and thereafter, mRNA expression levels of all cell cycle factors showed a decreasing trend and reached their lowest levels in 1–3 h, except for cyclin-dependent kinase 1 (CDK1) and CDK4 mRNA. The mRNA expression levels of CDK1, p21, and p27 increased markedly after stimulation with Ang II for 24–48 h. In myocardium tissue, CDK and cyclin expression levels peaked in 3–7 days, followed by a decreasing trend, while those of p21 and p27 mRNA remained at a high level on day 21. Expression levels of all protein were consistent with the results of mRNA in NRVMs or mice. The influence of Ang II or Iso on protein expression was more obvious than that on mRNA. TP treatment effectively prevented the imbalance in the expression of cell cycle regulators in the hypertrophy model group. In Conclusion, an imbalance in the expression of cell cycle regulators occurs during cardiac hypertrophy, and triptolide corrects these abnormal expression levels and attenuates cardiac hypertrophy.

## Introduction

Cardiac hypertrophy is considered to be a compensatory adaptation in response to intrinsic and extrinsic stimuli and is characterized by the reactivation of fetal genes such as β-myosin heavy chain (β-MHC) and brain natriuretic peptide (BNP), enhanced protein synthesis, increased sarcomere organization, and an increase in heart mass with the enlargement of cardiomyocytes (Tan et al., 2011; Pillai et al., 2015; Martens et al., 2020). Although it initially occurs as a compensatory response, a sustained increase in hypertrophy eventually leads to ventricular dilatation and heart failure (Pillai et al., 2015), which increases the morbidity and mortality of various cardiovascular diseases. Therefore, reversing pathological myocardial hypertrophy has become an important strategy for the prevention and treatment of cardiovascular diseases.

Cardiomyocytes in mammals proliferate speedily during embryonic stage but withdraw from the cell cycle soon after birth and are considered terminally differentiated cells. Accumulated studies have revealed that adult cardiomyocytes are capable of cell cycle reentry (Li et al., 2013; Zebrowski and Engel, 2013; Estrella et al., 2015). The cell cycle is controlled by various regulators, including cyclins, cyclin-dependent kinases (CDK) and CDK inhibitors (CDKI). Cell cycle regulators in cardiomyocytes can be reactivated and expressed when stimulated by pathologic factors. Therefore, cardiomyocytes can undergo DNA and protein synthesis but cannot complete mitosis successfully, resulting in an increase in mass and volume, i.e., cardiac hypertrophy (Ahuja et al., 2007). Although the myocardial expression and activation of main cyclin/CDK complexes, such as cyclin A-CDK1/2, cyclin B-CDK1, cyclin D-CDK4/6, and cyclin E-CDK2, remain at high levels during early mammalian embryonic stages (Tane et al., 2014), the protein expression of cell cycle regulators is significantly downregulated in cardiomyocytes after birth (Ahuja et al., 2007). Various researchers have indicated that cell-cycle regulation plays an important role during the hypertrophic growth of cardiomyocytes (Hinrichsen et al., 2008). Di Stefano et al. reported that triple transfection with p21, p27, and p57 siRNAs induced both neonatal and adult cardiomyocytes to enter the S phase and increased the nuclei/cardiomyocyte ratio, which implies that CDKI expression plays an active role in maintaining cardiomyocyte withdrawal from the cell cycle (Di Stefano et al., 2011). Moreover, CDKI downmodulation increased both cyclin E and A levels. Hence, cell cycle activity is not only required for cell division but also involved in hypertrophic growth. Considering that all the cell cycle regulators vary dynamically and play roles synergistically with the progression of cardiac hypertrophy, it is necessary to elucidate the expression pattern of the cell cycle regulator family and explore the possible mechanisms of the hypertrophic response.

Triptolide (TP), the major active component of the Chinese medicinal herb *Tripterygium wilfordii* Hook F, has been used for the treatment of some inflammatory, autoimmune disorders, such as lupus, nephrotic syndrome, Behçet’s disease, and rheumatoid arthritis, and a variety of cancers (Pan, 2010; Fan et al., 2016; Ziaei and Halaby, 2016; Hou et al., 2019; Noel et al., 2019). It has been suggested that TP could downregulate the expression of the retinoblastoma protein (Rb), cyclin A, cyclin B, CDK1, and CDK2; trigger cell cycle arrest in the S phase; and induce apoptosis in the cells involved in human renal cell carcinoma and multiple myeloma (Li et al., 2011; Noel et al., 2019). TP can also abrogate the growth of colon cancer and induce cell cycle arrest by inhibiting the transcriptional activation of early gene 2 promoter binding factor (E2F) (Oliveira et al., 2015). Besides the immunomodulatory and anti-cancer effects, It has been also reported that TP treatment can protect rat hearts against pressure overload-induced cardiac fibrosis, improve ventricular function (Wen et al., 2013; Zhang et al., 2013; Li et al., 2017) and attenuate cardiac hypertrophy (Ding et al., 2016), even though it may cause acute myocardial damage, such as myocardium denaturation, swelling, and necrosis when administered with high dose (Hou et al., 2019). However, the effects of TP on cell cycle regulators in myocardial hypertrophy and its mechanism are currently poorly understood. Therefore, the aims of the present study were to explore the temporal expression of cell cycle regulators in cardiac hypertrophy and the effects of TP *in vitro* and *in vivo*.

## Materials and Methods

### Animals

Seventy-two male C57BL/6J mice (18–21 g) were provided by the Experimental Animals Center of the Third Military Medical University (Chongqing, China). Animals were kept in the facility for 1 week to become accustomed to the new environment. Mice were randomly divided into model groups and TP groups, injected subcutaneously with isoproterenol hydrochloride (Sigma, St. Louis, MO, USA) 10 mg/kg twice daily to induce ventricular hypertrophy referring to the method described by other researchers (Lin et al., 2008; Ma et al., 2011; Taglieri et al., 2011). Animals in the model groups (isoproterenol-treated group, Iso) and TP groups were respectively injected intraperitoneally with vehicle or TP (purity 99.69%; Beijing Medicass Biotechnol, Beijing, China) at doses of 10 µg/kg daily for 0, 1, 3, 7, 14, or 21 days (*n* = 6 in each group). TP was dissolved in DMSO and then diluted in normal saline. The final used concentration of TP and DMSO was 1 μg/ml and 0.1% (v/v), respectively. All animals were weighed every 3 days, and the doses were adjusted accordingly. The mice in all groups were housed in an animal room with a 12 h light/12 h dark conditions and fed freely standard chow and water. The temperature and relative humidity were kept constant. All protocols conform to the *Guide to the Care and Use of Laboratory Animals* published by the Canadian Council on Animal Care and were approved by the Ethical Committee for Animal Experimentation of the Third Military Medical University.

### Sampling

At the end of the treatment, all animals were weighed. The mice were decapitated, and the hearts were carefully isolated, placed in a dish with normal saline, blotted slightly and then weighed. The tibial length (TL) was measured to calculate the ratio of heart weight and left ventricular weight to tibial length (HW/TL and LVW/TL, respectively), and the degree of ventricular hypertrophy was assessed by measuring the HW/TL ratio. The ventricles were divided into multiple segments for histology, RNA extraction, and total cellular protein extraction. Middle ventricular slice was fixed with a formalin neutral buffer solution, and the apex of the ventricle was collected and stored in liquid nitrogen for further experiments.

### Cell Culture and Treatment

Neonatal rat ventricular myocytes (NRVMs) from 1- to 3-day-old Sprague-Dawley rats were isolated and grown in DMEM containing 10% fetal bovine serum (FBS) and 1 × 10^-7^ mol/ml 5-Bromo-2-deoxyuridine under 5% CO_2_ air at 37°C for 48 h. NRVMs were cultured in serum-free DMEM for 24 h and then incubated for 0, 5, 10, 30 min, and 1, 2, 3, 6, 12, 24, 48 h, respectively, in a serum-free medium containing 1.0 μmol/L Ang II, to which 1.0 μg/L TP was added to determine its effects. The cell size was determined with rhodamine-phalloidin staining and analyzed with ImageJ software (NIH Image, National Institutes of Health, Bethesda, MD, USA).

### Cell Cycle Analysis

NRVM cells were cultured for 48 h in 6-well plates (5 × 10^6^ cells per well) and then cultured in serum-free DMEM for 24 h. After incubation for a scheduled time (0, 5, 10, and 30 min, and 1, 2, 3, 6, 12, and 24 h) in a serum-free medium containing 1.0 μmol/L Ang II, the cells were trypsinized, centrifuged (4°C, 2500 × g, 5 min), fixed in ice-cold 70% ethanol for 24 h, washed with PBS and centrifuged (10 min, 2500 × g, 4°C). The cells were incubated with 50 μg/ml RNase at 37°C for 30 min, stained with 50 μg/ml propidium iodide (PI) at room temperature in the dark for 20 min, and analyzed with flow cytometry to detect the cell cycle, and progression of the cell cycle was assessed.

### Real-Time PCR

Total RNA was extracted from NRVMs and left ventricular tissue using TRIzol reagent (TaKaRa, Japan) according to the manufacturer’s instructions. RNA was reverse transcribed with a Prime Script RT reagent kit (TaKaRa, Japan) into complementary DNA (cDNA) in a total volume of 20 μl. Real-time PCR for β-MHC, cyclin A1, A2, B1, D1, E1, CDK1, 2, 4, 6, and CDK inhibitor p21 and p27 mRNA were performed using primers designed by Premier 5.0 (Premier Biosoft International, Palo Alto, CA, USA) (**Table 1**). The reaction was performed in a total volume of 10 µl with QPK-201 SYBR Green PCR Master Mix (Bio-Rad, USA), and the thermal cycling conditions were as follows: after an initial 3 min at 95°C, the samples were subjected to 44 cycles comprising 10 s at 95°C for denaturation, 30 s at 58°C for annealing, and 30 s at 72°C for elongation, and finally, 10 min at 72°C for ending the reaction. All results were repeated in six independent experiments. The Ct values were normalized to both the β-actin expression levels and the normal controls, and the relative quantification was calculated using the 2^-ΔΔCt^ method.

**Table 1:** Primers used for real-time RT-PCR.

Species	Gene	Forward (5’→3’)	Reverse (5’→3’)
Rat	CDK1	TCTTCGCTCGTTAAGAGTTAC	ATCTGCCAGTTTGATTGTTC
	CDK2	GTTGACGGGAGAAGTTGTGG	GCTTGACGATGTTAGGGTGAT
	CDK4	CTACGGACATACCTGGACAAA	AATCTAGGCCGCTTAGAAACT
	CDK6	GTGGAAGTTCAGACGTGGAT	CAAGCATTTCAGAAGGAGGT
	cyclin A1	TCTGACCGTTCCAACCAC	TGAATAGCCCGTAAATGC
	cyclin B1	CAGGCTTTCTCCGATGTGAT	TGCTCTTCCTCCAGTTGTCTG
	cyclin D1	GCCCTCCGTTTCTTACTTCA	CTCCTCTTCGCACTTCTGCT
	cyclin E1	GTCAACGACACGGGAGAAGT	AGCAGCGAGGACACCATAAG
	p21	GGTGATGTCCGACCTGTTCC	ACGCTCCCAGACGTAGTTGC
	p27	TTGGGTCTCAGGCAAACTCT	GCAGGTCGCTTCCTCATCC
	β-MHC	GACAACGCCTATCAGTACATG	CCAATGGCAGCAATAACA
	β-actin	CGTAAAGACCTCTATGCCAACA	TAGGAGCCAGGGCAGTAATC
Mouse	CDK1	GCTTTTCCACGGCGACTCAG	ATCCAAGCCGTTCTCGTCCA
	CDK2	GCATTCCTCTTCCCCTCATCA	TCCAAAGGCTCTTGCTAGTCCA
	CDK4	GCTGCTACTGGAAATGCTGACC	TCACTCTGCGTCGCTTTCCT
	CDK6	TTCCAAATCTGCTCAACCCATC	CTGGTTGGATGGCAGGTGAG
	cyclin A2	GAGGCAGCCAGACATCACTAACA	AACACAGACATGGAGGAGAGGAAT
	cyclin B1	TCTCCATGCTGGACTACGACAT	AGCAGGGAGTCTTCACTGTAGGAT
	cyclin D1	GTGAGGAGCAGAAGTGCGAAGA	CTCGGCAGTCAAGGGAATGGT
	cyclin E1	GAAGGCCCTTAAGTGGCGTCT	AGCACCTCACCCGTGTCGTT
	p21	TACTTCCTCTGCCCTGCTGC	TGGTCTGCCTCCGTTTTCG
	p27	GCGGTGCCTTTAATTGGGTC	AGCAGGTCGCTTCCTCATCC
	β-MHC	TGCCAATGACGACCTGAAAGA	CGCTCGCTGGTCTCAATCAG
	β-actin	CCGTAAAGACCTCTATGCCAACA	CTGCTGGAAGGTGGACAGTGAG

### Western Blot Assay

NRVMs were stimulated with 1.0 μmol/L Ang II and 1.0 μg/L TP for 0.5, 2, 12, and 24 h, respectively. Total proteins were extracted from NRVMs or left ventricular tissue using RIPA lysis buffer (Beyotime, Shanghai, China) containing protease inhibitors. Equal amounts of denatured proteins were separated with 10% sodium dodecyl sulfate-polyacrylamide gels and then transferred to polyvinylidene difluoride membranes (Millipore, Bedford, MA, USA), which were blocked with 5% (w/v) nonfat milk powder in phosphate-buffered saline (PBS) containing 0.1% (v/v) Tween 20. Then, the membranes were incubated with primary antibodies for β-actin, β-MHC (1:500; Santa Cruz Biotechnology, USA), CDK4, CDK6, and cyclin D1 (1:500; Santa Cruz), and P21 and P27 (1:200; Santa Cruz) at 4°C overnight, followed by horseradish peroxidase (HRP)-conjugated secondary antibody. Antibody-reactive proteins were detected by means of chemiluminescence, and the intensity was captured using a ChemiDoc touch system (Bio-Rad, CA, USA).

### Morphological Observation of Myocardium

Left ventricular tissue was fixed in 4% formalin for 48 h, embedded in paraffin and cut into 5 μm slices. The left ventricular tissue slices were stained with hematoxylin/eosin (HE) staining, Masson staining, and fluorescein isothiocyanate (FITC)-labeled lectin wheat germ agglutinin (Sigma, St. Louis, MO, USA). The cross-sectional areas (CSAs) were measured in a blinded fashion with the Image Pro Plus 5.1 Image analysis program (Media Cybernetics, Silver Spring, MD, USA). The fraction of myocardial volume occupied by fibrillar collagen was calculated as the ratio of the fibrotic area to the total LV area.

### Statistical Analysis

The results are presented as the mean ± standard error of the mean (SEM), analyzed by one-way analysis of variance (ANOVA) with least significant difference (LSD) *post hoc* analyses with statistical software (SPSS, Chicago, IL, USA) and GraphPad Prism version 5.01 (GraphPad Software, La Jolla, CA, USA). *P* value of <0.05 was considered statistically significant.

## Results

### Ang II Induces Cell Cycle Re-Entry in NRVMs

**Figure 1:**
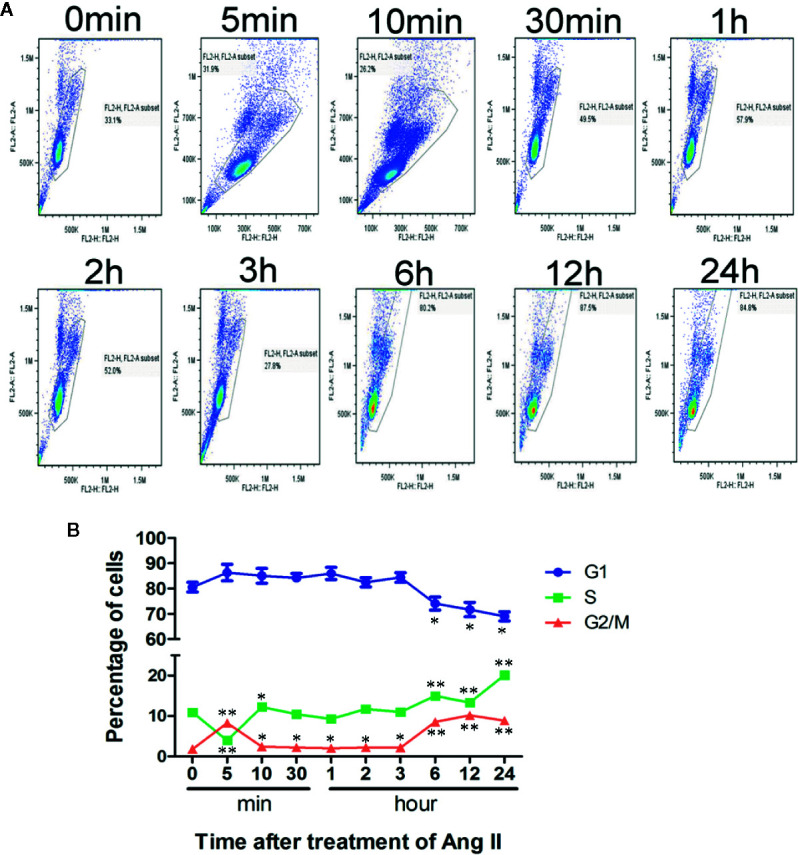
Cell cycle analysis after stimulation with Ang II. Neonatal rat ventricular myocytes (NRVMs) were treated with Ang II (1.0 µmol/L) for the scheduled times (0, 5, 10, and 30 min, and 1, 2, 3, 6, 12, and 24 h). **(A)** Flow cytometry analysis of cardiomyocytes stained with propidium iodide (PI). All experiments were conducted in duplicate and repeated three times (n = 3). **(B)** Data are presented as the mean ± SEM, ^*^P < 0.05, ^**^P < 0.01 compared with the controls (0 min), (one-way ANOVA).

To further confirm that pathological stimuli could promote cardiomyocyte re-entry into the cell cycle during cardiac hypertrophy, cell cycle analysis by flow cytometry with PI staining revealed significant cell cycle alterations in NRVMs after stimulation with Ang II. After stimulation for 5 min, the number of treated cells in the G_1_ phase increased more quickly than that of the controls (86.09% vs. 80.9%). Noticeably, the number of cells in the S phase showed a significantly increase after stimulation for 5 min. With the increase in stimulation time, the number of cells in the G_1_ phase showed a decreasing trend, and simultaneously, the S+G_2_ phase cell numbers increased. After Ang II stimulation for 24 h, we observed that 69.63% of NRVMs were found in the G_1_ phase, and 28.33% were found in the S+G_2_ phase. These results indicate that the NRVM cell cycle can be reinitiated by disease-related hypertrophic stimuli, which is represented by the increased cardiomyocyte number in the S+G_2_ phase while the number of cells in the G_1_ phase decreased significantly compared with that in the control group (**Figures 1A, B**).

### Hypertrophic Response of NRVM

**Figure 2:**
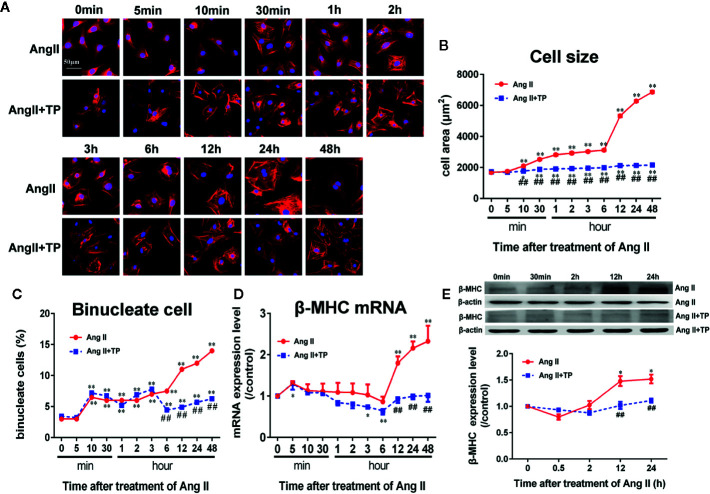
Triptolide attenuated the hypertrophic response of neonatal rat ventricular myocytes. **(A)** NRVMs treated with Ang II (1.0 µmol/L) and 1.0 μg/L TP for the scheduled times and stained with rhodamine-phalloidin (bar = 50 µm). **(B)** Cell size (*n* = 50 cells in each group). **(C)** Binucleate cell percentage (n = 6 analysis and 150 cells for each analysis). **(D)** mRNA expression of β-MHC was determined using real-time PCR (*n* = 6). **(E)** β-MHC protein expression was determined using Western blotting (*n* = 4). The data are presented as the mean ± SEM, ^*^P < 0.05, ^**^P < 0.01 compared with the controls (0 min), ^##^ *P < *0.01 compared with the Ang II-treated group at the same time point (one-way ANOVA).

The NRVM size increased soon after stimulation with Ang II (1.0 μmol/L), and the surface area of the NRVMs was significantly increased after stimulation for 10 min. Then, the surface area continued to increase with the addition of stimulation time. After stimulation for 24–48 h, the cell area significantly increased by 2.7–3.1-fold compared to the control (Ang II vs. control: 6285~6870 ± 61~71 vs. 1682 ± 35 µm^2^, respectively, p < 0.01). We observed that the TP treatment decreased cell size significantly compared with Ang II treatment (**Figures 2A, B**). Simultaneously, the number of binucleate cells elevated markedly after stimulated with Ang II, and TP treatment reduced it strikingly from 6 h (shown in **Figure 2C**).

Moreover, β-MHC mRNA and protein expressions, one of markers of cardiac hypertrophy and is induced by hypertrophic stimulation of NRVMs, was consistent with the results of cell size. The results showed that the mRNA and protein expressions of β-MHC did not alter significantly during the early stage of Ang II stimulation (within 3 and 12 h, respectively); however, during the late stage of cardiac hypertrophy, the mRNA and protein expression levels of β-MHC were the highest compared to those in the control group (0 min). Noticeably, TP significantly decreased the expression of β-MHC protein and mRNA compared to Ang II, as shown in **Figures 2D, E**.

### Expression Levels of Cell Cycle Regulators in NRVMs

**Figure 3:**
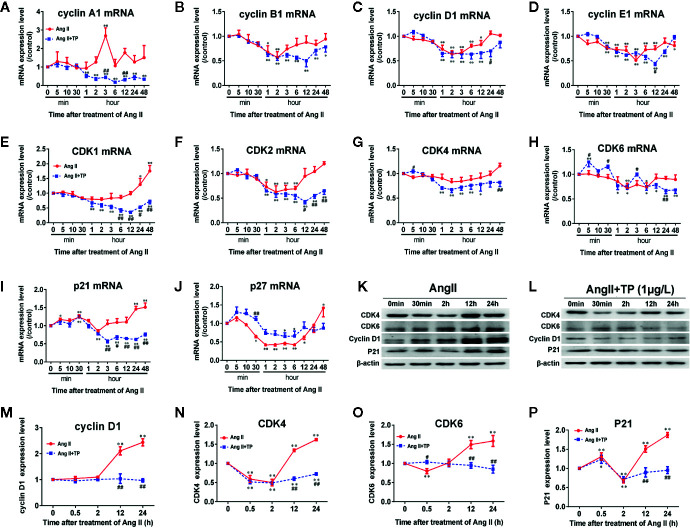
Expression of cell cycle regulators at the mRNA and protein levels in NRVMs. **(A–J)** The mRNA expression levels of cyclin, CDK, and CDKI were measured with real-time PCR (*n* = 6) and normalized to β-actin and the control group, respectively. **(K, L)** Cyclin D1, CDK4 and 6, and P21 protein levels as shown by Western blot analysis (*n* = 4). **(M–P)** The intensity of each band was quantified by densitometry and normalized to the β-actin and control groups. Data are shown as the mean ± SEM, ^*^P < 0.05, ^**^P < 0.01 compared with the controls (0 min), *^#^P < *0.05, *^##^P < *0.01 compared with the Ang II-treated group at same time point (one-way ANOVA).

Cell cycle regulators have been implicated in cardiomyocyte proliferation and cell cycle processes. Herein, we detected the mRNA expression of the cell cycle regulators cyclin A1, B1, D1, and E1; CDK 1, 2, 4, and 6; p21 and p27 using real-time PCR. The results showed that the mRNA expression of cyclin A1, p21 and p27 increased soon after stimulation with Ang II (1.0 μmol/L) for 5 min, and p21 mRNA expression reached its maximum at 30 min. The mRNA expression of all cell cycle factors showed a decreasing trend. The mRNA expression levels of CDK1, 2, 4, and 6; cyclin B1 and D1; and p21 were lowest at 2 h, and the mRNA expression levels of cyclin A1 and p27 mRNA reached their lowest at 1 h. Cyclin E1 mRNA had minimum expression at 3 h, and a transient elevation of the cyclin A1 mRNA level was confirmed at 3 h (**Figure 3A**). Only the expression of CDK1, p21, and p27 mRNA increased significantly after stimulation with Ang II for 24–48 h (*P*<0.01 or 0.05). The results demonstrated that the mRNA expression levels of cell cycle regulators fluctuate in the hypertrophic progression of cardiomyocytes. The TP treatment not only significantly decreased NRVM size but also effectively prevented the abnormal expression of cell cycle regulators compared to the vehicle (Ang II) in NRVMs (**Figures 3A–J**). To further investigate the effect of TP on the protein expression of cell cycle regulators in NRVMs, we examined the expression of several cell cycle-specific proteins, cyclin D1, CDK4 and 6, and P21, on cardiac hypertrophy progression. The results demonstrated that all protein expression levels were upregulated in cells stimulated with Ang II for 12–24 h compared with the controls. In contrast, TP treatment dramatically inhibited Ang II-induced protein expression compared with the vehicle. The mRNA analysis results were consistent with the results obtained in the protein expression analyses. Taken together, these results suggested that cell cycle regulators were abnormally expressed in the process of cardiomyocyte hypertrophy and that TP could balance the expression of cell cycle regulators and attenuate cardiac hypertrophy (**Figures 3K–P**).

### Survival of Mice

No animal died during the whole experimental period. All of the mice in the Iso groups showed waxy, lackluster fur, breathlessness and reduced activity, drumble, and lags in response with the stimulation time. Mice in the TP-treated groups were more active than those in the Iso group.

### Myocardial Hypertrophy

**Figure 4:**
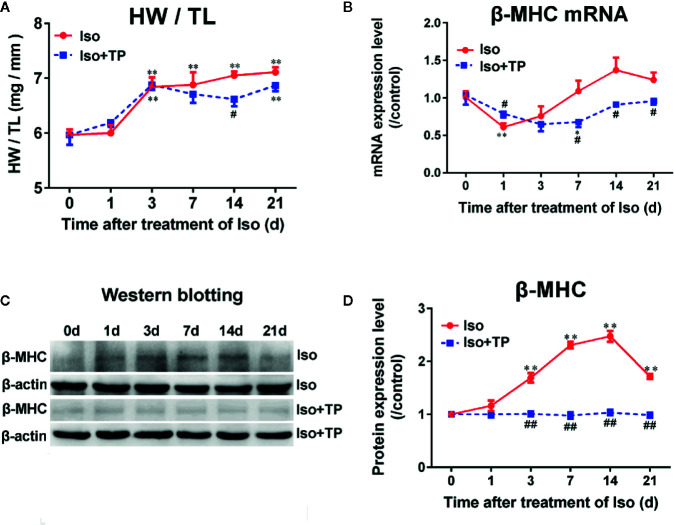
Triptolide attenuated cardiac hypertrophy in mice. **(A)** Heart weight (HW) index to tibia length (TL). **(B)** β-MHC mRNA expression level (*n* = 6). **(C)** β-MHC protein level as assessed by Western blot analysis (*n* = 4). **(D)** The intensity of each band was quantified by densitometry and normalized to β-actin and the control group. Data are shown as the mean ± SEM, ^*^P < 0.05, ^**^P < 0.01 compared with the controls (0 day); *^#^P <* 0.05, *^##^P <* 0.01 compared with the Iso-treated group at the same time point (one-way ANOVA).

First, the effects of TP on myocardial hypertrophy induced by Iso were observed. As shown in **Figure 4A**, mice showed a significant increase in heart weight (HW) to tibia length (TL) ratio compared with the normal control group after treatment with Iso for 3 days (*P* < 0.01). Then, the heart indexes (HW/TL) continued to increase with time. However, TP treatment could significantly decrease the heart index and inhibit the occurrence of myocardial hypertrophy compared with Iso groups, and the effect of inhibition was more obvious at day 14 (*P* < 0.01). Further studies showed that the mRNA expression of β-MHC was lowest at day 1, followed by an increasing trend (**Figure 4B**). Moreover, the protein level of β-MHC increased from day 0, and β-MHC expression levels of protein and mRNA reached a plateau at day 14, followed by a decreasing trend (**Figures 4B–D**). The results showed a compensatory response in mice. However, interestingly, the β-MHC protein displayed a more remarkable increase in myocardial tissue compared with mRNA during myocardial growth and hypertrophy. TP treatment downregulated β-MHC protein expression significantly (vs. Iso groups, *P* < 0.05). Collectively, these results indicate that TP treatment has the ability to inhibit the abnormal expression of β-MHC and attenuates the stimulation of pathologic factor-induced hypertrophy responses *in vivo*.

### Histological Findings

**Figure 5:**
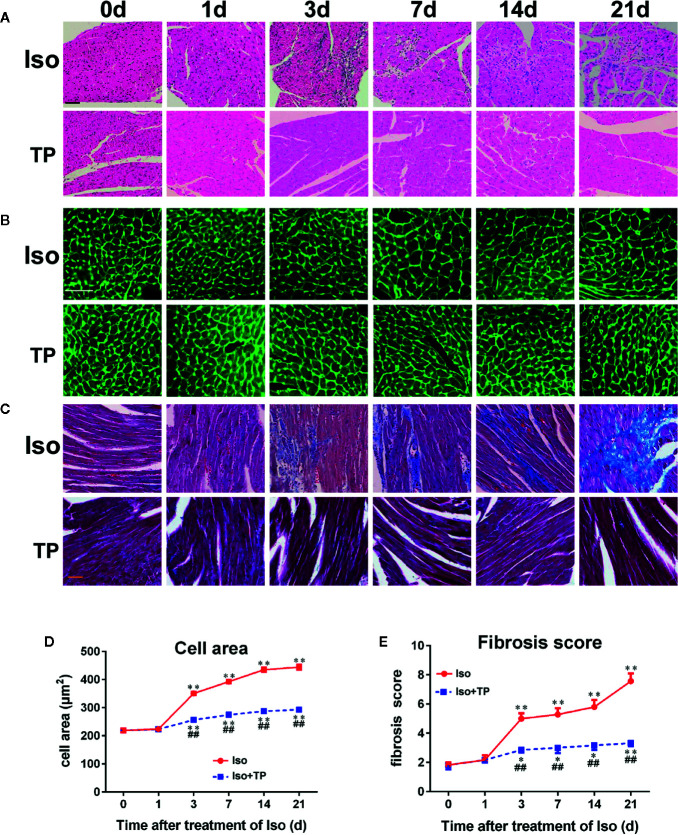
Myocardial histological changes after TP treatment. **(A)** Hematoxylin and eosin (HE) staining of myocardial LV tissue (bar = 50 µm). **(B)** Wheat germ agglutinin lectin staining (bar = 50 µm). **(C)** Masson’s trichome-stained left ventricular tissue (bar = 50 µm). **(D)** Cross-sectional area (*n* = 80). **(E)** Fibrosis score. Data are shown as the mean ± SEM (*n* = 6 in each group), ^*^P < 0.05, ^**^P < 0.01 compared with the controls (0 day); *^##^P < *0.01 compared with the Iso-treated group at the same time point (one-way ANOVA).

We next tested whether TP treatment attenuated myocardial hypertrophy using HE, lectin, and Masson staining. As shown in **Figure 5**, the histology of mice was almost normal at day 1 after Iso induction. However, we found a hypertrophic myocardium, myocardial fiber disruption, focal necrosis and inflammatory cell infiltration at day 3 after Iso induction, and myocardial injury was aggravated with stimulation time. The Iso groups revealed extensive myocardium degeneration and inflammatory cell infiltration at day 21 (**Figure 5A**). Furthermore, Iso stimulation resulted in a significant increase in the CSA of the myocardium and LV interstitial fibrosis compared with the control group (**Figures 5B, C**). The Iso group showed wide areas of collagen deposition, and the CSA significantly increased, near 1-fold, compared to the control at day 21 (*P <* 0.01). TP-treated mice showed a marked decrease in the degenerated myocardium and cellular infiltrations. TP inhibits Iso-induced inflammatory factor activation and thereby contributes to the attenuation of the myocardial pathological injury response in mice. TP treatment significantly decreased myocardial tissue damage, CSA and the fibrosis score compared with the Iso group (**Figure 5**). Collectively, these results suggest that TP treatment attenuated the abnormal pathological features of myocardial hypertrophy.

### Myocardial Expression of the Cell Cycle Regulators

**Figure 6:**
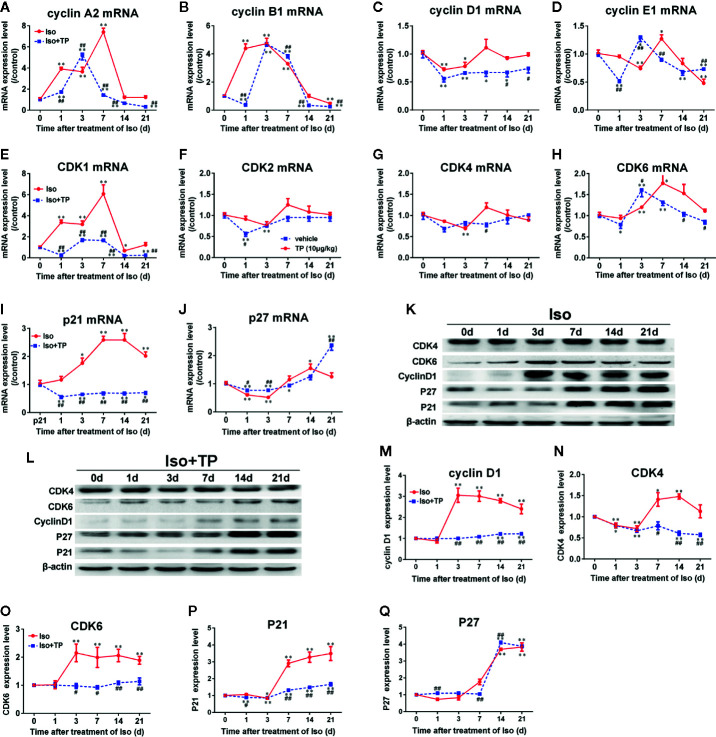
The effects of TP on the myocardial expression of cell cycle regulators in mice. **(A–J)** The mRNA expression levels of all cell cycle regulators. **(K, L)** cyclin D1, CDK4 and 6, P21, and P27 protein levels as shown by Western blot analysis (*n* = 4 per group). **(M–Q)** The intensity of each band was quantified by densitometry and normalized to β-actin and control group. The data are shown as the mean ± SEM (*n* = 5), ^*^P < 0.05, ^**^P < 0.01 compared with the controls (0 day); *^#^P < *0.05, *^##^P < *0.01 compared with the Iso-treated group at the same time point (one-way ANOVA).

Because the expression levels of cell cycle regulators in cardiomyocyte hypertrophy were abnormal, cell cycle regulators could play important roles in hypertrophy development. Therefore, we investigated whether pathological stimulation could induce abnormal expression of cell cycle regulators *in vivo*. The myocardial hypertrophic response of mice was induced by Iso. As shown in **Figure 6**, the Iso induced a gradual decrease in the myocardial mRNA expression of cyclin D1 and E1 and CDK2, 4, and 6 in mice. The mRNA expression of cyclin D1 and CDK6 reached a valley at day 1 and that of cyclin E1, CDK2 and CDK4 reached a valley at day 3. Following an increasing trend, the mRNA expression of cyclin A2, B1, and CDK1 increased dramatically on day 1, and cyclin B1 mRNA peaked on day 3. The expression levels of other factors all peaked at day 7, followed by a decreasing trend. After 21 days of treatment, the mRNA expression levels of cyclin B1 and E1 in the Iso groups were decreased markedly compared with those in the control group. Compared with the control, Iso upregulated the expression of p21 and decreased p27. The p27 mRNA expression level reached the lowest point on day 3 and increased to peak on day 14 with the expression of the p21 mRNA (**Figures 6A–J**). The protein expression levels of cell cycle regulators were determined using Western blotting. The results demonstrated that the expression levels of cyclin D1 and CDK6 increased dramatically post-Iso induction and reached a peak on day 3, followed by a decreasing trend. The protein expression levels of CDK4 and P21 were lowest on day 3, followed by a dramatic increase, and CDK4 reached a peak on day 14. P21 and P27 showed a significant, nearly 4-fold increase in the Iso group compared to the control group at day 21 (*P <* 0.01). However, TP treatment could effectively correct abnormal expression of the cell cycle regulators cyclin, CDK, and CDKI (**Figures 6K–Q**).

## Discussion

Cardiomyocyte death is a common end-point in many forms of cardiovascular disease, and a number of approaches have been explored to increase cardiomyocyte number in injured hearts, with the hope of promoting functional recovery after myocardial infarction (Hassink et al., 2008). It is believed that cardiomyocyte cell cycle activation is of great value (Pasumarthi and Field, 2002; Hassink et al., 2008; Eghbali et al., 2019). For decades, the common dogma was that the adult heart is incapable of regenerating lost myocardium after injury. The present knowledge suggests that cardiomyocytes in the adult mammalian heart exhibit some capacity to re-enter the cell cycle, but they cannot successfully complete mitosis, which results in cardiac hypertrophy (Ahuja et al., 2007). These issues, namely, that evidence of DNA and cell cycle protein synthesis, re-expression of embryonic genes or even nuclear division in cardiac myocytes does not necessarily represent true cardiac myocyte division, have been the major hurdles in the field when interpreting results (Beltrami et al., 2001). Cell cycle activity in adult cardiac myocytes can represent multinucleation and polyploidization (DNA replication without karyokinesis or cytokinesis) or DNA repair in addition to true proliferative activity. However, it is potentially even more problematic in patients with diseased hearts where cardiac myocyte ploidy increases dramatically with a patient’s diastole and systole (Zhang et al., 2015). Cardiac hypertrophy has been regarded as an independent risk factor for cardiovascular morbidity and mortality (Cox and Marsh, 2014). Myocardial hypertrophy and fibrosis are the predominant pathological changes in myocardial remodeling, which results in both diastolic and systolic dysfunction (Li et al., 2017). In the present study, we found that NRVM size increased soon after stimulation with Ang II. Simultaneously, the number of binucleate cells increased with time. The overall change trends of cell size, binucleate cells percentage, and β-MHC are similar although the change of binucleate cells is not exactly the same as the expression of β-MHC mRNA. Flow cytometry analysis indicated that the number of myocytes in the S+G_2_ phases increased and that in the G_1_ phase decreased significantly compared with the control group. Significant cell cycle alterations were observed in NRVMs during cardiac hypertrophy. The cariomyocytes response quickly to neurohumoral stimulators, such as Ang II and Iso. Ang II activates extracellular signal regulated kinase (ERK) and other MAPKs, as well as immediate-early gene, such as c-fos, which promote DNA synthesis. It has been reported that the increase of c-fos expression and ERK activation occur as quick as 2 min after Ang II stimulation (Sadoshima and Izumo, 1993; Aoki et al., 2000).

Cell cycle machinery driven by cyclin and CDK activity, is positively regulated by growth factors and negatively regulated by CDKI families. These CDKIs, which form complexes with cyclin–CDK, exert negative effects. The human genome encodes 21 CDKs, although only seven (CDK1-4, 6, 10, and 11) have been shown to have a direct role in cell cycle progression. Other CDKs play an indirect role *via* the activation of other CDKs, regulation of transcription or neuronal function (Malumbres et al., 2009; Sánchez-Martínez et al., 2015). In addition, it has been reported that the human genome contains 29 genes encoding cyclins (Malumbres and Barbacid, 2005). Of these, CDK1, 2, 4, and 6 and A, B, D, and E-type cyclins are identified as the major regulators of the cell cycle and are related to cell cycle progression (Bretones et al., 2015). Key regulators of G_1_ progression are mainly cyclin D, which associates with and activates its catalytic partner CDK4, and CDK6 which phosphorylates the retinoblastoma protein (pRb), thereby activating E2F transcription factors. Cyclin E is mainly expressed at the G_1_/S transition, and it binds to and activates CDK2 to accelerate the phosphorylation of the Rb proteins, which is required for entry into the S-phase and for DNA synthesis. Cyclin A/CDK2 plays a critical role in the control of the S phase and DNA replication and is also essential for G_2_ progression. Cyclin A- and cyclin B-associated CDK1 regulates the G_2_/M phases (Ahuja et al., 2007; Dai et al., 2013). Cell cycle activity is dependent on the balance between active and negative regulators (Zhang et al., 2015). The miR-16 inhibits the hypertrophic phenotype in cardiomyocytes through downregulation of CCND1, CCND2, and CCNE1 expression and inactivation of the cyclin/Rb pathway (Huang et al., 2015). Similar results were also seen in cyclin A2-overexpressing transgenic mice, including the induction of cardiac regeneration, reduced scarring, improved LV function, and prevention of heart failure after myocardial infarction (Cheng et al., 2007). Cyclin A2 induces cardiac regeneration and improves cardiac function after myocardial injury (Shapiro et al., 2014). Cardiomyocyte cell cycle activation improves cardiac function after myocardial infarction (Hassink et al., 2008).

TP is an extract of the Chinese herb TwHF, which is widely used in China to treat autoimmune and inflammatory diseases due to its anti-inflammatory and immunosuppressive effects. TP attenuates pressure overload-induced myocardial remodeling in mice *via* the inhibition of NLRP3 inflammasome expression (Li et al., 2017). Additionally, in recent years, studies have suggested that TP could attenuate cardiac hypertrophy by upregulating FOXP3 expression and ameliorating cardiac fibrosis (Zhang et al., 2013; Ding et al., 2016). However, few studies have examined the regulatory role of TP in cardiomyocyte cycle regulator expression in cardiac hypertrophy.

Our results revealed that cyclin A1, p21, and p27 mRNA were highly expressed in NRVMs after stimulation with Ang II for 5 min. After 30 min, the mRNA expression levels of all cell cycle regulators showed a decreasing trend and reached their lowest levels at 1–3 h. Cell cycle analysis indicated that the number of myocytes in phases G_1_ and G_2_ was decreased compared with the 5 min group, which is related to the inhibition of the cell cycle. The expression of cell cycle regulators recovered later. Additionally, the number of myocytes in the S and G_2_ phases increased and that in the G_1_ phase decreased, demonstrating cell cycle reentry. However, only the expression of CDK1, p21, and p27 mRNA increased significantly after stimulation with Ang II for 24–48 h. The results showed the abnormal expression of cell cycle regulators with time during the process of hypertrophic response. Furthermore, a similar phenomenon occurs in adult myocytes; after treatment with Iso for 3–7 days, the expression levels of cell cycle regulators peaked, followed by a decreasing trend. After 21 days of Iso treatment, the mRNA expression levels of cyclin B1 and E1 were decreased markedly compared with those in the control group. In the present study, we found that the levels of the cell cycle inhibitors P21 and P27 were highest in the end stage of cardiac hypertrophy, whereas the cell cycle-promoting factors cyclins and CDK returned to the levels seen in normal adult myocytes. Interestingly, it has been shown that the effects of Ang II or Iso on protein expression were more obvious compared to effects on mRNA. Taken together, our results revealed abnormal expression of cell cycle regulators at both the mRNA and protein levels in NRVMs and adult mouse ventricles during cardiac hypertrophy. These findings indicate that cell cycle regulators are involved in cardiomyocyte hypertrophy. However, TP treatment attenuates the hypertrophy of cardiomyocytes through the effective correction of the abnormal expression of the cell cycle regulators cyclin, CDK, and CDKI. After treatment with Iso, from day 3 to day 21, heart index (HW/TL) and myocardial CSA increased significantly in the Iso group compared with the control group (0 day). Morphological analysis showed that myocardial injury, inflammatory cell infiltration and collagen deposition were markedly aggravated with increasing time in Iso-treated animals. Nevertheless, there was no significant between-group difference in the liver or kidney. The weights of the lung showed an increasing trend and were significantly increased in the Iso group compared with the control group after treatment with Iso for 21 days (data not shown). TP treatment could effectively prevent the abnormal expression of cell cycle regulators and markedly decrease the heart index, improve tissue injury and attenuate myocardial fibrosis compared with Iso groups.

**Figure 7:**
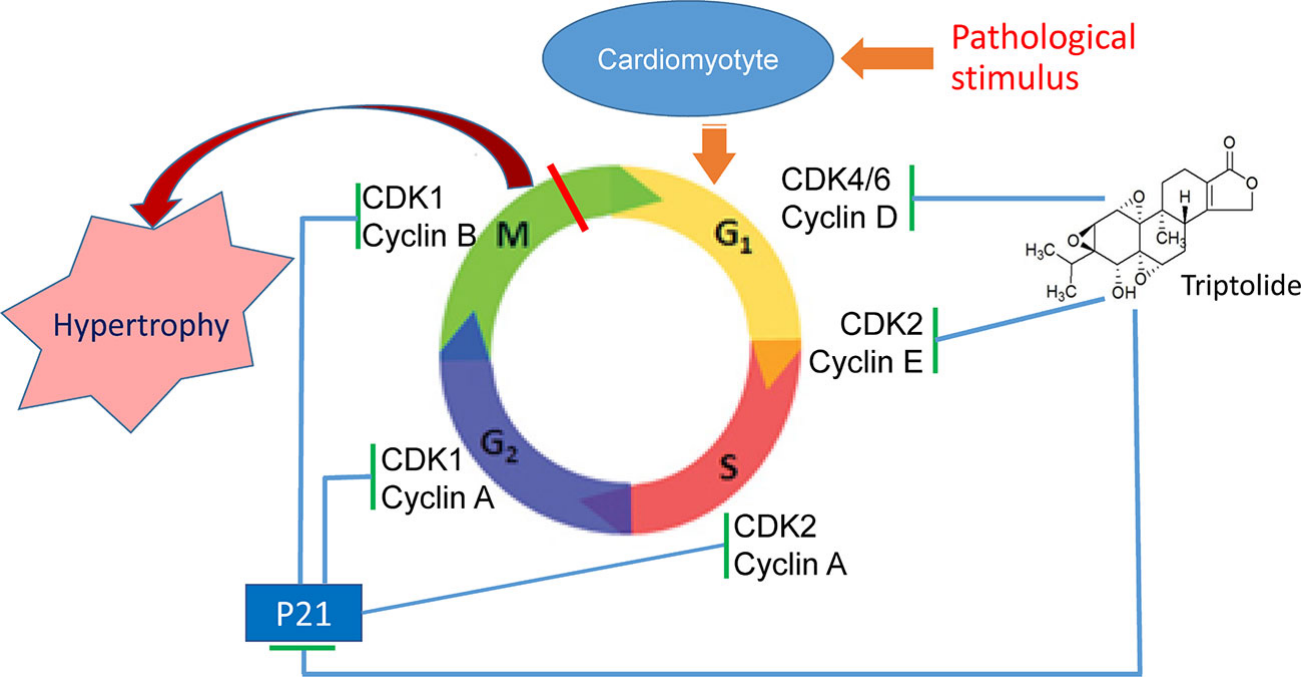
Triptolide (TP) ameliorates cardiac hypertrophy *via* correcting the abnormal expression of cell cycle regulators in cardiomyocytes.

The data in the current study indicate that the alteration of expression of cell cycle regulators is closely related with cardiac hypertrophy. Our study demonstrated that TP can mitigate pathological stimulation-induced cardiac hypertrophy and effectively alleviate the myocardial damage response *via* regulation of myocardial cell cycle regulators. In this study, we found that pathological cardiac hypertrophy in NRVMs and mice is associated with abnormal expression of β-MHC and the cell cycle regulators cyclin, CDK, and CDKI in cardiomyocytes and the myocardium. TP treatment significantly inhibits the occurrence of myocardial hypertrophy, reduces the left ventricular index, ameliorates pathological alterations by correcting the abnormal expression of various cell cycle regulators (**Figure 7**). Collectively, our results indicated that TP treatment had regulatory effects on cardiac hypertrophy induced by pathological stimulation and potentially acted through the inhibition of the abnormal expression of various cell cycle regulators, which is altogether indicative that the regulation of the cell cycle could become an important strategy for the prevention and treatment of cardiac hypertrophy.

Our study still has certain limitations. Although it has been found that the use of low dose of TP alone had no effects on the cell size of cariomyotytes *in vitro* and *in vivo* (Tong, 2015; Ding, 2017), its influence on the cell cycle of normal myocytes need to be explored. And the exact mechanism underlying the regulation of cell cycle regulators remains to be elucidated.

In summary, our results preliminarily revealed the temporal dynamic expression pattern of cell cycle regulators in cardiomyoctes during cardiac hypertrophy and demonstrated TP has the potential to attenuate cardiac hypertrophy *via* correction of the abnormal expression of various cell cycle regulators.

## Data Availability Statement

The raw data supporting the conclusions of this article will be made available by the authors, without undue reservation, to any qualified researcher.

## Ethics Statement

The animal study was reviewed and approved by the Ethical Committee for Animal Experimentation of the Third Military Medical University.

## Author Contributions

H-GZ designed the experiments, drafted and revised the manuscript, and prepared the final version of the manuscript. X-HC and YL revised and prepared the final version of the manuscript. J-ML, Y-YD, YL, X-CP, and Y-FT performed the experiments and analyzed and interpreted the data. All authors contributed to the article and approved the submitted version.

## Funding

This work was supported by grants from the Natural Science Foundation of Chongqing, China (No. cstc2018jcyjAX0504), and the Special project for promoting scientific and technological innovation capability: Frontier exploration project, Third Military Medical University (No. 2019XQY05).

## Conflict of Interest 

The authors declare that the research was conducted in the absence of any commercial or financial relationships that could be construed as a potential conflict of interest.

## Abbreviations

Ang II, angiotensin II; CSA, cross-sectional area; CDK, cyclin-dependent kinase; CDKI, cyclin-dependent kinase inhibitor; HW, heart weight; Iso, isoprenaline; LVW, left ventricular weight; MHC, myosin heavy chain; NRVM, neonatal rat ventricular myocyte; TP, triptolide.
